# Effects of different milk feeding levels and frequencies on performance of Holstein heifers during weaning and first lactation

**DOI:** 10.1038/s41598-022-22560-y

**Published:** 2022-10-22

**Authors:** Fatemeh Ahmadi, Majid Akbarian-Tefaghi, Azam jafari, Morteza Hosseini Ghaffari

**Affiliations:** 1 Department of Animal Sciences, College of Agriculture, Isfahan University of Technology, Isfahan, 84156–83111 Iran; 2 Department of Animal Science, Faculty of Agriculture, Lorestan University, PO Box 465, Khorramabad, 68151-44316 Iran; 3 Institute of Animal Science, University of Bonn, 53115 Bonn, Germany

**Keywords:** Physiology, Developmental biology

## Abstract

In the present study, the effects of milk feeding amount and feeding frequency (FF) on performance, blood metabolites, rumen fermentation, and performance of dairy heifers during the first artificial insemination (AI) and first lactation were investigated. The treatment group consisted of 48 female Holstein heifer calves (12/treatment) distributed in a 2 × 2 factorial arrangement with milk feeding [high = 8 L/d, HL or medium = 6 L/d, ML] and feeding frequency [2 × or 3 ×]. All calves were fed on a step-up/step-down method, weaned from milk at 56 d of age, and observed until 70 d of age. Heifer calves were kept uniform from 70 d of age until the first lactation to evaluate the long-term effects of the treatments. We observed no FF effect and no interaction between the level and FF on feed intake, average daily gain (ADG), feed efficiency (FE), body weight (BW), structural growth, blood metabolites, and rumen fermentation parameters. Calves fed HL milk had higher total dry matter intake (DMI), BW, and ADG during the pre- and post-weaning periods. During the preweaning period, HL-fed calves had higher serum insulin (trend) and glucose concentrations compared to ML-fed calves. Lower age at first AI was observed in calves fed HL rather than ML regardless of FF. Weight at first AI, milk yield (305 d), and energy-corrected milk (ECM-305 d) in the first lactation showed a trend toward higher values in HL-fed calves compared to ML-fed calves. Preweaning performance and calf maturity can be positively affected by feeding high amounts of milk at both FF.

## Introduction

The purpose of giving calves a large volume of milk or milk replacer (MR) during the preweaning period is to promote weight gain during the preweaning period and future calf performance^1,2^. In addition, raising calves with high milk or MR content has generally been shown to improve calf well-being by reducing signs of hunger^3,4^ and decreasing the incidence of disease, possibly due to better nutrition and improved immunity^5^. The disadvantage of higher milk or MR feeding is that it is associated with lower feed intake at weaning, lower rumen development, and lower growth in the wk before and after weaning^6,7^. Therefore, feeding high volumes of milk in step-up/step-down (SUSD) is a good strategy to increase solid feed intake in the last week before weaning and has a positive effect on solid feed intake and growth performance after weaning^8,9^. In the step-up/step-down method, the amount of milk for calves is gradually increased until the maximum amount is reached in the middle of the milk feeding period (at about 7 weeks of age), and then gradually decreased to the lowest level immediately before weaning to promote the intake of starter feed and to support rumen development and good growth^8^. Heifer performance is strongly influenced by preweaning ADG, and higher preweaning weight gain may reduce calving age, first artificial insemination (AI), gestation age, and age at first calving and improve first lactation performance^10,11^.

In addition to the amount of milk, the frequency of milk feeding is also important, especially when heifer calves receive a large amount of milk or MR. Farmers in the industry are trying to mimic natural conditions by using automatic milk feeders that allow calves to exhibit natural feeding practices in terms of quantity, frequency, and duration of milk meals^12,13^. However, there are still many farms that feed calves with buckets^14,15^ and that feed moderately to high quantities of milk in only two meals to save labor and costs^15^. This can lead to problems such as milk reflux into the reticulorumen due to rapid and high milk intake in one meal, which increases the risk of diarrhea and decreases the growth performance of dairy calves^16,17^. In addition, feeding large amounts of milk with limited frequency can lead to decreased intake of starter feed, delayed rumen growth^7,18^, lower digestibility of starter feed^18^, excessive gas formation, and abomasal bloat^19^. Therefore, the number of meals rather than their size should be increased in this situation.

Several studies have been conducted to determine the effect of feeding frequency (FF) on growth rate. According to Van den Borne et al.^16^, increasing FF from 2 to 4 meals per day at high feeding rates of energy and protein metabolism in heavy male calves (136 kg; 15 wk old) improved the efficiency of energy and protein utilization and thus animal growth. MacPherson et al.^20^ studied only the effect of FF on glucose and insulin kinetics and found that feeding large amounts of MR (8 L daily MR, 26% crude protein, and 18% fat) twice daily (meal size 4 L) compared with feeding it four times daily (meal size 2 L) resulted in lower insulin sensitivity and had no effect on calf growth rate. Another study examining FF and protein source in MR for calves concluded that feeding MR (with nipple buckets) 2 meals/day compared to 3 meals improved growth before weaning (weight gain, BW) but had no effect on growth after weaning^21^.

Studies that have examined the effects of FF with varying amounts of milk or MR are limited. For example, Jafari et al.^22^ examined the effects of FF in calves fed low and high volumes of milk (4 vs 10 l/d) and found that regardless of milk volume, feeding 2 meals/d instead of 3 meals/d resulted in increased intake of starters and increased total intake DM during the preweaning and postweaning periods and increased ADG during the preweaning period. Recently, another study by Orellana Rivas et al.^19^, examined two amounts MR [0.65 (low) or 0.76 (high) kg DM per day of 26% crude protein and 17% fat MR] and two FF (2 × or 3 ×). Results showed that increasing MR improved growth in both summer and winter, while increasing FF improved growth only in winter. According to Jafari et al.^22^, increasing milk feeding frequency from 2 to 3 times per day was not beneficial for calves fed low (4 L/d; total milk intake 210 L) or high (up to 10 L/d; total milk intake 371 L) amounts of milk, and calves fed low milk performed poorly because of low nutrient intake. In addition, feeding 3 meals per day reduced total DM and starter intake without affecting average daily gain, regardless of milk feeding levels^22^. Therefore, the present study examined medium and high milk volumes with different frequencies. Because of the reported benefits of feeding high milk volumes on animal performance^23^, the dairy industry has experienced an extreme shift from low to medium or high milk volumes in feeding.

Therefore, the objective of this study was to investigate the interaction between medium and high whole milk volumes (15% and 20% of BW, 6 and 8 l/d, respectively) and FF (2 × vs. 3 ×) with the step-up/step-down pattern and bucket feeding for short-term effects (performance, blood metabolites, rumen fermentation) and long-term effects (first breeding performance and milk production in the first lactation). We hypothesized that calf performance would improve due to a more even distribution of nutrients throughout the day when calves were fed a higher volume and frequency of milk.

## Materials and methods

Before the study’s onset, ethical approval for all methods involving animals was obtained from the Animal Care and Use Committee of the Isfahan University of Technology. All methods were carried out following Iranian Council of Animal Care regulations^24^. The study complies with ARRIVE guidelines for reporting in vivo experiments and all methods were performed in accordance with the relevant guidelines and regulations.

### Calves, management, and treatments

The study was conducted from February to April 2018 at Fazil Dairy Farm (Isfahan, Iran). Immediately after birth, calves were separated from their dams, weighed, and housed in individual pens (1.2 m × 1.5 m from birth to 3 d of age and 1.8 m × 2.8 m from 4 to 70 d of age) in the barn with natural ventilation. Wheat straw was used as bedding, which was replaced every 48 h. Calves received 3 l of high-quality colostrum (Brix value > 22) via nipple bottles at each of the first two feedings (i.e., within 30–60 min after birth and 8 h after the first feeding). From the second feeding until the third day of life, all calves received transition milk (4 l) twice a day at 0800 and 1600 h. The quality of the colostrum was assessed with a digital Brix refractometer (PAL-1, Atago Co. Ltd, Bellevue, WA, USA) and discarded if it had a value less than 22 on the Brix scale. Blood samples were obtained from the jugular vein 24 h after the first colostrum feeding by venipuncture with Clot Activator Vacutainers (BD Vacutainer, Franklin Lakes, NJ, USA). As an indicator of passive immunity transfer, total serum protein was measured with a handheld refractometer (VET 360; Reichert Inc, Depew, NY, USA). A serum protein level > 5.5 g/dL was required for calves to be included in the study.

**Figure 1:**
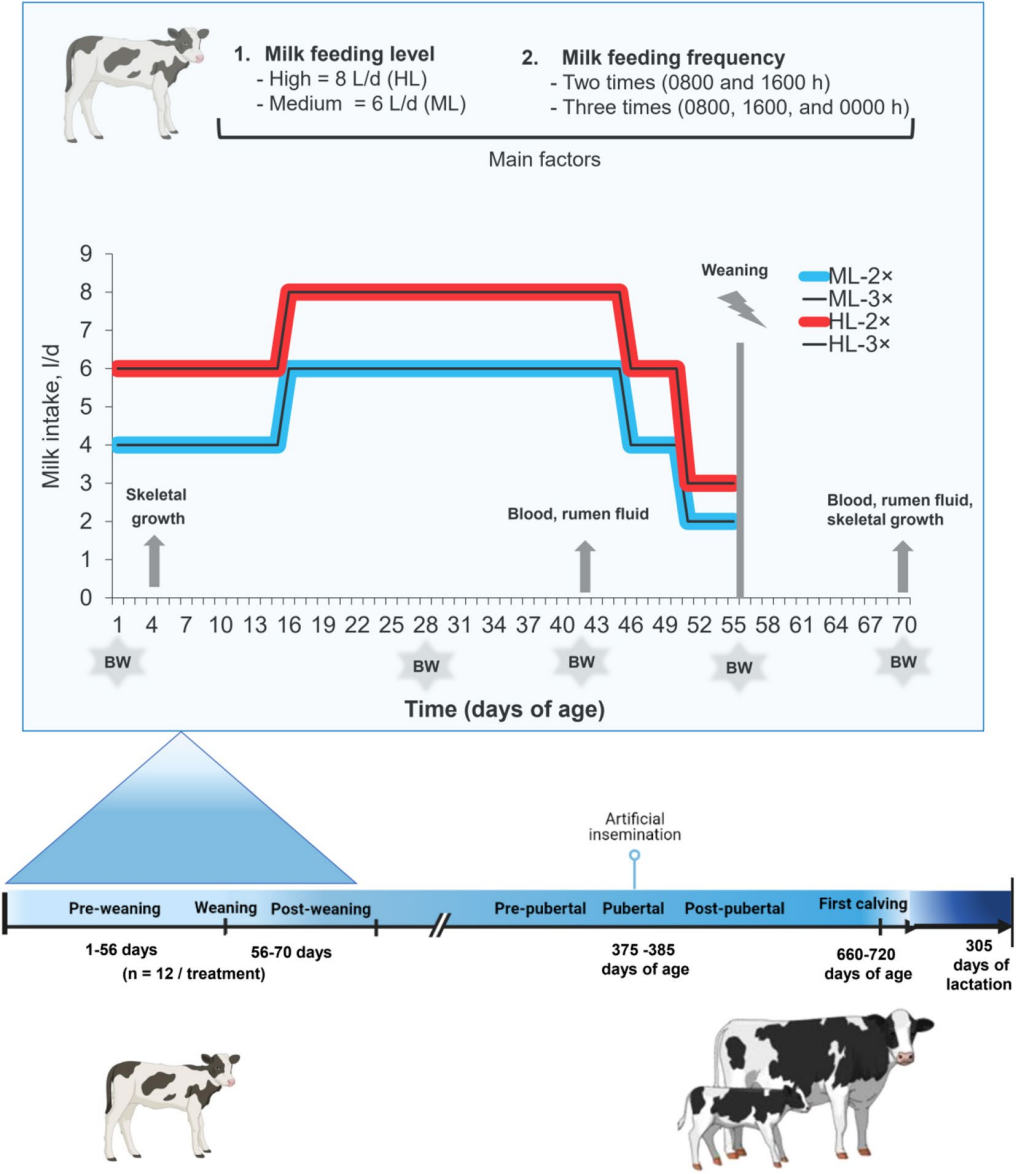
Overview of the animal experiment. The schematic diagram represents the amounts of milk consumed (L/d) by calves fed different amounts of milk and weaned at 56 d of the study. Calves fed medium milk allowance twice or thrice per day (2 × or 3 × ; 4 l/d of milk from d 1–15, 6 l/d of milk from d 16–45, 4 l/d of milk from d 46–50, and 2 l/d of milk from d 51–55 of the study); calves fed high milk allowance twice or thrice per day (2 × or 3 × ; 6 l/d of milk from d 1–15, 8 l/d from d 16–45, 6 l/d from d 46–50, 3 l/d from d 51–55 of the study). Figure created using BioRender (https://biorender.com/).

A total of 48 Holstein heifer calves (41.2 ± 1.5 kg body weight, n = 12 females per treatment) were randomly assigned to 4 treatments in a 2 × 2 factorial design (Fig. 1): two medium milk feedings (6 l/d and 2 meals/d, ML-2 × ; 6 l/d and 3 meals/d, ML-3 ×) and two high milk feedings (8 l/d and 2 meals/d, HL-2 × ; 8 l/d and 3 meals/d, HL-3 ×). Calves in the medium-level treatments received 4 l/d from d 1 to 15, 6 l/d from d 16 to 45, 4 l/d from d 46 to 50, and 2 l/d from d 51 to 55 (total milk intake = 260 l). Calves in the high milk feeding treatments received 6 l/d from d 1 to 15, 8 l/d from d 16 to 45, 6 l/d from d 46 to 50, and 3 l/d from d 51 to 55 (total milk intake = 360 l). All calves were fed pasteurized whole milk (60 °C for 30 min) twice (0800 and 1600 h) or three times (0800, 1600, and 0000) via bucket. Calves had free access to a textured calf starter and water throughout the experiment. Starter feed consisted of corn and barley in the form of steam flakes and pellets with other ingredients. The ingredients and nutrient composition of the starter feed are shown in Table 1. The starter feed offered was adjusted daily to achieve 5 to 10% of the orts (i.e., the portion of the starter feed not eaten within 24 h); orts were collected and weighed at 0800 h daily. Calves diagnosed with the disease were treated by a veterinarian according to the farm's standard operating procedures. All calves were weaned from milk at 56 d of age and observed until 70 d of age.

**Table 1:** Ingredients, and chemical composition of the experimental starter.

Item	Starter feed
**Ingredient, % of DM**	
Steam-flaked corn	42.0
Steam-rolled barley	18.0
Soybean meal (45% CP)	31.5
Full fat soybean	4.7
Calcium carbonate	0.75
Vitamin and mineral mix^2^	2.0
Sodium bicarbonate	0.5
Salt	0.55
**Chemical composition of starter (% of DM)**	
DM	90.1 ± 0.06
Crude protein (CP)	21.9 ± 0.33
Ether extract (EE)	3.4 ± 0.21
Ash	6.4 ± 0.07
NDF	15.2 ± 0.18
NFC^2^	48.8
ME^3^, Mcal/kg DM	3.21
Ca^3^	0.9
P^3^	0.5

^1^Contained per kg of supplement: Vit A (IU) = 1,300,000, Vit D3 (IU) = 360,000, Vit E (IU) = 12,000, Ca (g) = 240, Mg (g) = 67, Zn (mg) = 20,000, Cu (mg) = 4290, I (mg) = 200, Co (mg) = 100, Mn (mg) = 10,000, Se (mg) = 80. ^2^Nonfiber carbohydrates = 100 − (% NDF + % CP + % ether extract + % ash) (NRC^25^).

^3^Calculated from NRC^25^.

Heifer calves were kept uniformly from 70 d of age until the first lactation. We collected data on age at first AI, withers height at first AI (WH), weight at first AI, age at first calving, service per conception, 305-d milk yield, energy-corrected milk for 305-d yields (ECM-305 d), 305-d fat and protein yield of 305 d, and lactation length (Fig. 1). Heifers were kept in group pens (5 heifers/pen) with straw bedding from 70 to 120 days of age and then moved to pens with compost bedding for 60 heifers at 4 months of age. Heifers had ad libitum access to a TMR that met the nutritional requirements of growing Holstein heifers at a gain of 0.9 kg BW per day based on NRC recommendations^25^. Heifers were not bred until they reached the desired criteria (minimum 340 kg BW and 128 cm withers height (WH)), but not before 12.5 months of age. Heifers were weighed and WH was measured monthly until the first AI service before morning feeding. After the first calving, primiparous cows were kept separately from multiparous cows in the herd in free stalls with sand bedding. Feed was offered three times daily in the form of a TMR based on the nutrient requirements of lactating cows with a milk yield of 40 kg/day according to the NRC^25^. Cows were milked 3 × daily (at 0600, 1400, and 2200 h) and milk production and milk composition were recorded for individual animals via the Animal Center of Isfahan Agricultural Jahad Organization and Vahdat (Isfahan, Iran) during the first lactation (305 DIM). In addition, energy-corrected milk for 305-d yields (ECM-305 d) was calculated using the following equation: ECM at 305 DIM (kg) = [(0.327 × kg milk) + (12.95 × kg milk fat) + (7.20 × kg milk protein)], according to Carvalho et al.^26^.

### Chemical analysis

Throughout the experiment, samples of starters and orts were collected daily and pooled weekly for analysis. Until analysis, starter samples were stored at − 18 °C. We determined the DM content of the samples after drying them in an oven for 48 h at 65 °C. The Willey mill (Arthur Thomas Co. Philadelphia, PA) was then used to grind the samples to pass through a 1-mm sieve. The ground samples were analyzed for CP, ether extract, and ash using the methods of AOAC International^27^ and for NDF using heat-stable α-amylase^28^. Daily samples of bulk milk were collected and analyzed with Milkoscan (MilkoScan 134 BN, FossElectric, Hillerod, Denmark). The composition of the milk contained 3.45 ± 0.11% fat, 2.98 ± 0.04% CP, and 12.2 ± 0.12% total solids.

### Body weight and body measurement

Calves were weighed at the beginning of the study (birth) and at 28, 42, 56, and 70 d of age using an electronic scale (model EES-500; Ettehad Inc., Isfahan, Iran) that was calibrated before the beginning of the study and every month thereafter. Average daily gain (g/d) was calculated as the difference between BW measured in different periods divided by the number of d between periods at the individual level. Feed efficiency (FE) was calculated as g ADG/g total DMI (liquid feed (DM) + DMI of starter feed). Body measurements included body length (distance from shoulder to rump), withers height (distance from base of front feet to withers), body girth (distance around belly over part of back), hip height (distance between base of hind feet and hook bones), heart girth (circumference of rib cage), and hip width (distance between tips of hook bones) of calves, which were also recorded at 4 and 70 d of age, as described by Khan et al.^29^.

### Blood and ruminal fluid samples

Blood samples were collected from the jugular vein at the ages of 42 and 70 d by venipuncture with the Clot Activator Vacutainer (Vacutest®, Arzergrande, Italy). After collection of the samples, the tubes were immediately placed on ice and centrifuged at 2,500 g for 20 min at 4 °C. The separated serum was stored at − 20 °C until analysis. Serum glucose concentration was determined using commercial kits (Pars Azmoon Co., Tehran, Iran) with an automated biochemical analyzer (Technicon RA1000; Bayer Corp., Tarrytown, NY, USA). Serum concentrations of insulin were measured with an ELISA kit (Monobind Inc., Lake Forest, CA). The intra- and inter-assay coefficients of variation for insulin measurement were 6.9% and 8.2%, respectively. Blood concentrations of B-hydroxybutyrate (BHB) and blood urea nitrogen (BUN) were determined using the Autoanalyzer and commercial kits (Randox Laboratories Ltd, Ardmore, UK, and Parsazemun Co, Karaj, Iran).

On days 42 and 70 of the experiment, rumen fluid samples were collected by a stomach tube 4 h after the morning feeding and strained through four layers of cheesecloth. Rumen pH was immediately determined using a portable digital pH meter (HI 8318; Hanna Instruments, Cluj-Napoca, Romania) calibrated with pH 4.0 and 7.0 buffer solutions. Four ml of rumen fluid was acidified with 1 ml of 25% metaphosphoric acid and stored (− 20 °C) until analysis for VFA. Rumen samples were analyzed for VFA by gas chromatography (model CP-9002, Chrompack, Middelburg, The Netherlands) using a 50 m (0.32 mm i.d.) fused silica column (CP—Wax Chrompack Capillary Column, Varian, Palo Alto, CA, USA) with crotonic acid (1:7, vol/vol) as the internal standard, as described by Bal et al.^30^. Helium was used as the carrier gas, and the initial and final oven temperatures were 55 and 196 °C, respectively. The detector and injector temperatures were set at 251 °C.

### Statistical analysis

Based on previously published values^31,32^, a standard deviation of 100 g ADG per day was assumed, and the minimum meaningful difference in ADG was set at 65–75 g per day. From the power test analysis with α = 0.05 and power (1 − β) = 0.80, the predicted sample size was 12 calves per treatment for growth performance, which was considered the most reliable parameter to determine power. Data were analyzed as a completely randomized design with a 2 × 2 factorial arrangement of treatments with milk quantity (high vs. medium) and FF (2 vs. 3 times). Data were analyzed using the MIXED procedure from SAS (SAS 9.4, SAS Institute Inc., Cary, NC) with ANOVA, where time served as a repeated measure of starter feed intake, total DMI, ADG, FE, body measurement, rumen fermentation characteristics, and blood metabolites during the overall period, with the individual calf as the experimental unit. The model included fixed effects of milk level, milk feeding FF, time (d or wk), and their interactions. The calf was included as a random effect. Before analysis, all data were tested for normality using the UNIVARIATE procedure of SAS (Shapiro–Wilk test). Data that did not meet the assumptions of homoscedasticity and normality of residuals had to be log-transformed (base 10). Data were tested for heteroscedasticity using Levene's test in the statistical JASP software (JASP Team, 2019). Three variance–covariance structures (autoregressive type 1, compound symmetry, and Toeplitz) were tested, and an autoregressive type 1 covariance structure was selected as the best fit based on the Bayesian information criterion. The main effects of milk level, milk FF, and interactions were tested using ANOVA. Body weight, first and final body measurements, rumen fermentation, and blood metabolites at 42 and 70 days were analyzed using the above model without the influence of time. Initial BW was used as a covariate for the final weight. First-breeding performance (age, weight, and wither height at first AI, age at first calving, and service per conception) and lactation variables including milk yield, fat yield, protein yield, and lactation length were analyzed using PROC MIXED from SAS (SAS 9.4, SAS Institute Inc., Cary, NC). The statistical model included milk level treatment, FF treatment, and milk level × FF as fixed effects and heifers as random effects. The Tukey–Kramer adjustment was applied to account for multiple comparisons. The significance threshold was set at *P* ≤ 0.05; trends were declared at 0.05 < *P* ≤ 0.10.

## Results

### Intake and growth performance

**Figure 2:**
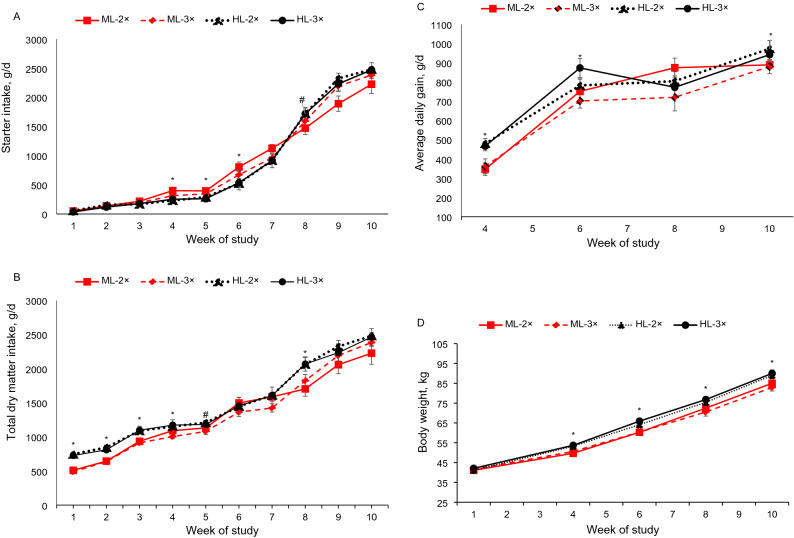
Starter feed intake (**A**), total dry matter intake (**B**), average daily gain (**C**), and body weight (**D**) of calves with one of the following treatments: calves fed medium milk level and 2 × frequency (medium-2 × ; red filled square), calves fed medium milk level and 3 × frequency (medium -3 × ; red filled rhombus), calves fed high milk level and 2 × frequency (High-2 × ; black filled triangle), and calves fed high milk level and 3 × frequency (High3 × ; black filled circle) during the different week of study. Error bars represent the SEM at each point. For each time point, *denotes significant differences (*P* < 0.05), # denotes trend differences (0.05 < *P* < 0.10) for level milk effects.

Data on starter intake, total DMI, ADG, and FE are shown in Table 2. Calves fed HL consumed 100 l more milk during the milk feeding period than calves from ML (360 l vs. 260 l), and all calves did not refuse milk. No significant interaction was observed between the level of milk feeding and FF for starter feed intake (g/d) during the preweaning or postweaning and overall periods. Although starter feed intake before weaning and overall was not affected by milk amount, the interaction milk amount × time (*P* < 0.01) showed that starter feed intake was greater in calves receiving ML than in those receiving HL from 4 to 6 wk of age (Fig. 1A). However, HL calves tended to increase starter feed intake in the postweaning period but not in the preweaning period. Mean total DMI (g/d; starter DM + milk DM) showed no differences between treatments (Table 2; Fig. 2B). Regardless of FF, mean DM intake was greater in HL-fed calves before weaning (1267 vs 1115 g/d; *P* < 0.01) and throughout the study period (1488 vs 1335 g/d; *P* < 0.01) than in ML-fed calves, and a trend (*P* = 0.09) was observed in the postweaning period. In addition, the interaction between milk amount and time was significant for total DMI during the preweaning period (*P* < 0.01), and HL-fed calves consumed more DM than ML-fed calves (*P* < 0.01) during wk 1 to 5 (trend, *P* = 0.06; at wk 5; Fig. 1B).

**Table 2:** Effects of milk feeding level (medium vs. high) and milk feeding frequency (2 vs. 3) on starter intake, total DMI, ADG, feed efficiency of dairy calves during preweaning, postweaning, and overall (n = 12 calves/treatment).

Item	Treatments^1^				SEM	*P*-value^2^						
	ML-2 ×	ML-3 ×	HL-2 ×	HL-3 ×		L	F	L*F	T	L*T	F*T	L*F*T
**Starter DIM g/d**												
Preweaning	592	546	508	504	44.2	0.16	0.57	0.63	< 0.01	< 0.01	0.56	0.18
Postweaning	2142	2288	2402	2349	86.0	0.09	0.52	0.17	< 0.01	0.87	0.66	0.88
Overall	902	894	887	870	38.5	0.61	0.75	0.91	< 0.01	< 0.01	0.89	0.64
**Total DMI g/d**^**3**^												
Preweaning	1138	1092	1269	1265	44.2	< 0.01	0.57	0.63	< 0.01	0.01	0.56	0.18
Postweaning	2142	2288	2402	2349	86.0	0.09	0.52	0.17	< 0.01	0.87	0.66	0.88
Overall	1339	1331	1496	1480	38.4	< 0.01	0.75	0.91	< 0.01	0.23	0.89	0.64
**ADG, g/d/calf**												
Preweaning	658	595	689	704	29.2	0.02	0.41	0.18	< 0.01	0.10	0.16	0.34
Postweaning	890	880	973	942	33.1	0.03	0.54	0.74	-	-	-	-
Overall	716	666	760	763	22.5	< 0.01	0.31	0.24	< 0.01	0.16	0.27	0.35
**Feed efficiency**^**4**^												
Preweaning	0.52	0.51	0.52	0.54	0.02	0.25	0.98	0.69	< 0.01	< 0.01	0.23	0.42
Postweaning	0.44	0.39	0.41	0.42	0.02	0.92	0.39	0.21	< 0.01	0.87	0.66	0.88
Overall	0.50	0.49	0.50	0.51	0.01	0.23	0.81	0.42	< 0.01	< 0.01	0.32	0.62
**BW, kg**												
Initial (d 4)	41.1	41.6	41.2	42.1	0.68	0.67	0.31	0.76	-	-	-	-
Final (d 70)	85.4	82.8	89.4	89.3	1.53	< 0.01	0.38	0.39	-	-	-	-

^1^Treatments including medium milk level- feeding 2 times (ML-2 ×), medium milk level- feeding 3 times (ML-3 ×), high milk level- feeding 2 times (HL-2 ×), and high milk level- feeding 3 times (HL-3 ×).

^2^Statistical comparisons: L = feeding level; F = feeding frequency, L × F = feeding level × feeding frequency, T = time, L × T = feeding level × time, F × T = feeding frequency × time, and L × F × T = feeding level × feeding frequency × time.

^3^Total DMI = DM intake of milk + starter.

^4^ Feed efficiency = ADG (g)/DMI (g).

Regardless of FF, ADG was higher in HL-fed calves than in ML-fed calves during preweaning (696 vs. 626 g/d; *P* = 0.02), postweaning (957 vs. 885 g/d; *P* = 0.03), and throughout the study periods (761 vs. 691 g/d; *P* < 0.01; Fig. 2C). Average daily gain was not affected by the interaction between milk feeding levels and FF. Feed efficiency did not differ between treatments. However, an interaction between milk quantity and time (*P* < 0.01) for feed efficiency showed that HL-fed calves had higher feed efficiency than ML-fed calves at 4 wk of age, but there was no difference thereafter. Body weights of calves fed HL were higher compared with calves fed ML at 28, 42, 56, and 70 d of age (Fig. 2D).

### Skeletal growth

The results of body measurements, including body length, heart girth, body barrel, withers height, hip height, and hip width at the beginning (d 4 of age) and end of the experiment (d 70 of age) are shown in Table 3. None of the body measurements were affected by milk feeding levels, FF, or their interactions, except for a trend in withers height, which was greater in HL calves than in ML calves at 70 d of age (93.9 vs. 92.8 cm; *P* = 0.08).

**Table 3:** Effects of milk feeding level (medium vs. high) and milk feeding frequency (2 vs. 3) on skeletal growth of dairy calves during days of 4, 70, and overall (n = 12 calves/treatment).

Item	Treatments^1^				SEM	*P*-value^2^						
	ML-2 ×	ML-3 ×	HL-2 ×	HL-3 ×		L	F	L*F	T	L*T	F*T	L*F*T
**Heart girth, cm**												
4 d	81.2	82.2	81.9	82.2	0.84	0.65	0.46	0.65				
70 d	102	101	101	102	1.82	0.69	0.73	0.48				
Overall	91.8	91.9	91.3	92.2	1.01	0.88	0.53	0.67	< 0.01	0.58	0.97	0.41
**Body barrel, cm**												
4 d	83.8	85.7	85.6	85.8	0.86	0.27	0.22	0.35				
70 d	115	115	116	116	1.75	0.72	0.86	0.98				
Overall	99.7	100	101	101	0.99	0.37	0.58	0.61	< 0.01	0.72	0.40	0.58
**Body length, cm**												
4 d	44.7	44.6	44.4	45.7	0.63	0.47	0.32	0.26				
70 d	54.3	54.8	53.9	55.2	0.74	0.95	0.24	0.61				
Overall	49.5	49.7	49.2	49.8	0.50	0.65	0.14	0.26	< 0.01	0.59	0.91	0.61
**Hip height, cm**												
4 d	82.9	83.2	82.2	82.7	0.79	0.46	0.60	0.83				
70 d	98.0	97.7	98.4	97.3	0.83	0.96	0.39	0.65				
Overall	90.4	90.4	90.3	90.0	0.56	0.61	0.85	0.90	< 0.01	0.53	0.35	0.65
**Hip width, cm**												
4 d	18.0	18.2	18.5	18.3	0.30	0.35	0.90	0.50				
70 d	25.0	24.8	25.3	25.0	0.35	0.47	0.50	0.81				
Overall	21.5	21.5	21.9	21.7	0.22	0.22	0.72	0.49	< 0.01	0.82	0.61	0.73
**Wither height, cm**												
4 d	78.9	78.5	79.2	78.2	0.80	0.95	0.38	0.71				
70 d	93.0	92.7	94.4	93.5	0.62	0.08	0.30	0.62				
Overall	85.9	85.6	86.8	85.8	0.55	0.41	0.23	0.62	< 0.01	0.25	0.86	0.99

^1^Treatments including medium milk level- feeding 2 times (ML-2 ×), medium milk level- feeding 3 times (ML-3 ×), high milk level- feeding 2 times (HL-2 ×), and high milk level- feeding 3 times (HL-3 ×).

^2^Statistical comparisons: L = feeding level; F = feeding frequency, L × F = feeding level × feeding frequency, T = time, L × T = feeding level × time, F × T = feeding frequency × time, and L × F × T = feeding level × feeding frequency × time.

### Blood metabolites parameters

Blood metabolites data are shown in Table 4. Serum concentrations of BHB and BUN metabolites were not affected by milk feeding levels, FF, and their interactions. The serum concentrations of glucose were greater in HL-fed calves than in ML-fed calves (120.5 vs 92.3 mg/dl; *P* = 0.03) at d 42 of age. Serum insulin concentrations tended to be higher (*P* = 0.07) in HL-fed calves than in ML-fed calves (17.9 vs. 13.8 µU/ml), but no differences were observed on d 70 of age. Regardless of the treatments, the concentrations of BUN and BHB increased and glucose concentrations decreased with age in all calves (*P* < 0.01).

**Table 4:** Effects of milk feeding level (medium vs. high) and milk feeding frequency (2 vs. 3) on blood metabolites of dairy calves during days of 42, 70, and overall (n = 12 calves/treatment).

Item	Treatments^1^				SEM	*P*-value^2^						
	ML-2 ×	ML-3 ×	HL-2 ×	HL-3 ×		L	F	L*F	T	L*T	F*T	L*F*T
**Glucose, mg/dl**												
d 42	93.5	91.2	119	122	9.60	0.03	0.81	0.63				
d 70	74.2	79.0	85.6	72.8	8.37	0.85	0.73	0.25				
Overall	83.9	85.1	102	96.6	6.89	0.20	0.65	0.53	< 0.01	0.17	0.99	0.22
**BHB, mmol/L**												
d 42	0.035	0.057	0.045	0.045	0.01	0.91	0.34	0.34				
d 70	0.277	0.350	0.392	0.327	0.04	0.36	0.94	0.18				
Overall	0.156	0.204	0.218	0.188	0.02	0.62	0.45	0.11	< 0.01	0.62	0.45	0.91
**Insulin, µU/ml**												
d 42	15.3	12.4	19.6	16.3	1.70	0.07	0.16	0.93				
d 70	17.6	19.6	17.1	17.9	2.00	0.64	0.54	0.81				
Overall	16.3	15.9	18.2	16.9	1.52	0.40	0.63	0.80	0.15	0.11	0.14	0.98
**BUN, mg/dl**												
d 42	21.2	22.6	20.4	19.5	2.02	0.37	0.91	0.60				
d 70	24.2	28.2	28.2	28.0	2.65	0.49	0.49	0.45				
Overall	22.7	25.4	24.3	23.6	1.81	0.95	0.60	0.38	< 0.01	0.23	0.58	0.80

^1^Treatments including medium milk level- feeding 2 times (ML-2 ×), medium milk level- feeding 3 times (ML-3 ×), high milk level- feeding 2 times (HL-2 ×), and high milk level- feeding 3 times (HL-3 ×).

^2^Statistical comparisons: L = feeding level; F = feeding frequency, L × F = feeding level × feeding frequency, T = time, L × T = feeding level × time, F × T = feeding frequency × time, and L × F × T = feeding level × feeding frequency × time.

### Ruminal fermentation parameters

Rumen fermentation data are shown in Table 5. Rumen total VFA concentration, the molar proportion of individual VFA, acetate-propionate ratio, and rumen pH were not affected by milk feeding levels, FF, and their interactions at 42 and 70 d of age. Total rumen VFA concentration and molar proportion of acetate, propionate, and butyrate were affected by calf age, with acetate decreasing while propionate and butyrate were higher at 70 d of age than at 42 d of age.

**Table 5:** Effects of milk feeding level (medium vs. high) and milk feeding frequency (2 vs. 3) on rumen fermentation of dairy calves during days of 42, 70, and overall (n = 12 calves/treatment).

Item	Treatments				SEM	*P*-value						
	ML-2 ×	ML-3 ×	HL-2 ×	HL-3 ×		L	F	L*F	T	L*T	F*T	L*F*T
**pH**												
d 42	5.24	5.85	5.91	5.78	0.21	0.20	0.31	0.12				
d 70	5.43	5.50	5.62	5.63	0.20	0.48	0.88	0.81				
Overall	5.34	5.68	5.77	5.70	0.15	0.16	0.38	0.21	0.32	0.57	0.45	0.29
**Total VFA, mmol/L**												
d 42	95.9	94.3	81.0	80.7	8.49	0.11	0.90	0.94				
d 70	104	99.8	102	105	3.47	0.56	0.84	0.36				
Overall	99.9	97.1	91.8	92.9	4.85	0.23	0.86	0.69	< 0.01	0.08	0.97	0.76
**Individual VFA, mol/100 mol**												
**Acetate**												
d 42	51.3	50.7	52.6	54.8	2.19	0.23	0.70	0.53				
d 70	46.7	45.1	45.6	44.8	3.04	0.79	0.65	0.83				
Overall	49.0	47.9	49.1	49.7	1.28	0.46	0.86	0.49	0.01	0.46	0.65	0.82
**Propionate**												
d 42	36.1	36.1	36.7	30.9	2.29	0.32	0.22	0.22				
d 70	40.6	38.9	38.1	40.4	1.57	0.76	0.84	0.22				
Overall	38.3	37.5	37.4	35.7	1.20	0.26	0.30	0.70	0.01	0.55	0.32	0.13
**Butyrate**												
d 42	8.07	8.35	7.14	8.68	0.67	0.66	0.20	0.36				
d 70	8.03	11.0	12.5	10.8	1.79	0.23	0.72	0.21				
Overall	8.09	9.66	9.84	9.76	1.21	0.42	0.30	0.56	< 0.01	0.10	0.95	0.08
**Valerate**												
d 42	4.02	3.97	2.79	4.64	0.53	0.59	0.12	0.09				
d 70	3.81	4.46	3.05	3.17	0.77	0.21	0.62	0.74				
Overall	3.93	4.21	2.92	3.91	0.51	0.22	0.24	0.50	0.57	0.40	0.57	0.16
**Isovalerate**												
d 42	0.51	0.86	0.79	0.99	0.18	0.28	0.17	0.70				
d 70	0.86	0.58	0.74	0.88	0.20	0.68	0.74	0.35				
Overall	0.68	0.72	0.76	0.94	0.09	0.15	0.31	0.49	0.90	0.72	0.33	0.42
**Acetate: propionate**												
d 42	1.45	1.43	1.48	1.80	0.15	0.21	0.34	0.29				
d 70	1.16	1.18	1.20	1.12	0.10	0.94	0.76	0.63				
Overall	1.30	1.30	1.34	1.46	0.06	0.13	0.34	0.34	< 0.01	0.40	0.45	0.36

^1^Treatments including medium milk level- feeding 2 times (ML-2 ×), medium milk level- feeding 3 times (ML-3 ×), high milk level- feeding 2 times (HL-2 ×), and high milk level- feeding 3 times (HL-3 ×).

^2^Statistical comparisons: L = feeding level; F = feeding frequency, L × F = feeding level × feeding frequency, T = time, L × T = feeding level × time, F × T = feeding frequency × time, and L × F × T = feeding level × feeding frequency × time.

### First-breeding and lactation performance

Data on first breeding performance, milk production, and milk quality of heifers (305 d) are presented in Table 6. Forty-two heifers (11, 10, 10, and 11 for HL-2 × , HL-3 × , ML-2 × , and ML-3 × , respectively) completed the first lactation. Wither height at first AI, age at first calving, and service per conception at first parity did not differ among treatments. Weight at first AI tended to be greater in HL-fed calves than in ML-fed calves (365 vs. 353 kg; *P* = 0.09). Calves fed more milk were inseminated earlier, and age at first AI was lower in HL-fed calves than in ML-fed calves (376 vs. 383 d; *P* = 0.05). Milk fat yield, milk protein yield (kg/d) in the first lactation, and lactation length were not affected by milk feeding levels, FF, and their interaction. However, a positive effect of feeding a high amount of milk during the preweaning period was observed on milk production during 305 d (*P* = 0.07) and ECM-305 d (*P* = 0.09) in the first lactation, which tended to be 1195 kg, and 1052 kg higher in HL-fed calves than in ML-fed calves, respectively.

**Table 6:** Effects of milk feeding level (medium vs. high) and milk feeding frequency (2 vs. 3) on first breeding and first lactation of heifers.

Item	Treatments^1^				SEM	*P*-value^2^		
	ML-2 ×	ML-3 ×	HL-2 ×	HL-3 ×		L	F	L*F
**First breeding performance**								
Wither height at AI^3^, cm	131	133	132	133	1.21	0.50	0.23	0.45
Weight at AI, kg	356	350	366	365	6.91	0.09	0.64	0.69
Age at first AI, d	384	382	377	375	3.70	0.05	0.51	0.88
Age at first calving, month	22.3	23.9	23.7	23.5	1.02	0.57	0.48	0.34
Service per conception (first parity)	2.0	1.8	1.8	2.4	0.56	0.96	0.72	0.50
**Milk yield and milk quality, kg**								
Milk 305 d, kg	10,350	10,747	11,127	12,360	556	0.07	0.20	0.51
ECM 305 d, kg	9435	10,007	10,129	11,418	593	0.09	0.13	0.55
Fat 305 d, kg	307	320	329	401	34.8	0.20	0.29	0.46
Protein 305 d, kg	289	326	309	304	16.3	0.94	0.33	0.21
Lactation length, d	396	343	304	308	53.6	0.19	0.53	0.87

^1^Treatments including medium milk level- feeding 2 times (ML-2 ×), medium milk level- feeding 3 times (ML-3 ×), high milk level- feeding 2 times (HL-2 ×), and high milk level- feeding 3 times (HL-3 ×).

^2^Statistical comparisons: L = feeding level; F = feeding frequency, L × F = feeding level × feeding frequency.

^3^AI = Artificial insemination.

## Discussion

The objective of this study was to determine the effects of the level of milk feeding and FF on calf performance, blood and rumen fermentation in calves, and the first lactation of dairy cows. Our results indicate that the level of milk feeding and FF do not interact to affect the growth performance of heifer calves. Therefore, the level of milk feeding and FF were discussed separately.

### Effect of milk feeding level

Intake of starter feed was low in the first 3 weeks of life in all treatments but was higher in calves fed ML than HL during the 4 to 6 wk when milk feeding peaked. This decrease in starter feed intake in HL-fed calves could be related to a strong negative correlation between liquid and starter feed intake^24^ (r = − 0.82), especially when the DMI for liquid feed was greater than 0.8 kg/d (DM intake of HL-fed calves in this study = 0.98 kg/d). In the current study, starter feed intake increased rapidly in all calves between wk 6 and 8 of the study when the amount of milk offered was reduced before weaning, and HL-fed calves tended to increase their starter feed intake at wk 8 and after weaning. Starter feed intake increased more than twofold in ML-fed calves and about threefold in HL-fed calves between wk 6 and 8 of the study, independent of FF. Apparently, at the time of weaning, the decrease in total nutrient intake caused by the loss of milk supply in calves is compensated for by a rapid increase in concentrate intake in dairy calves^18,33^. It is also possible that step-up/step-down weaning methods stimulated solid feed intake in calves with high milk content^9^. This procedure allowed calves to obtain more milk in the middle of the milk feeding period, while the gradual reduction in the amount of milk (10 days in this study) stimulated the intake of solid feed in the step-down phase. The use of a step-down weaning protocol has been shown to help increase solid food intake and maintain higher growth rates from pre- to post-weaning in calves fed high milk or MR^6,29,34^. According to our results, the transition from weaning with the step-up/step-down method was smooth for all calves, with starter feed intake for ML and HL calves not decreasing (1533 and 1719 g/d in the wk before weaning and 2042 and 2278 g/d in the wk after weaning) and no decrease in weight gain in the postweaning period. Consistent with the study by Klopp et al.^34^, we did not observe a large decrease in ADG in calves after weaning, which may be related to the small difference in DM intake between ML- and HL-fed calves (1505 and 1608 g/d, respectively).

In general, calves fed HL consumed more DM in the preweaning period and throughout the study and tended to consume more DM in the postweaning period than calves fed ML. The increase in DM intake during the pre-weaning period is related to higher milk intake (100 L) and also leads to an increase in starter consumption in HL-fed calves after weaning. In agreement with our results, other researchers have also found that an increase in milk consumption during the suckling period increases DMI in the preweaning period^8,35^. However, Klopp et al.^34^ observed no differences between calves fed medium and high amounts of MR in terms of intake of DM throughout the study period (1 to 56 d). Jafari et al.^22^ reported that DMI was higher in calves fed a high amount of milk before weaning than in calves fed a low amount of milk (10 L vs. 4 L/d). However, after weaning, DMI was higher with low milk volume than with high milk volume, probably due to the lower dependence of these calves on milk consumption compared with high milk volume (10 L/d).

Initial BW and skeletal growth did not differ among calves in the current study, indicating that all calves had similar structural measurements at baseline. An increase in BW at 28, 42, 56, and 70 d and ADG during preweaning, postweaning, and the entire period with feeding high levels of milk than moderate levels of milk indicates the advantage of accelerated milk over medium milk in improving growth performance. Consistent with studies by Orellana Rivas et al.^36^ and van Niekerk et al.^18^, ADG was higher in calves fed a high level of milk replacer during the preweaning period, likely due to greater milk replacer consumption containing a greater amount of digestible nutrients^34^. This is likely due to the higher milk consumption ingesting a greater amount of available nutrients^34^, and allowing calves to utilize greater amounts of nutrients for growth and development. Orellana Rivas et al.^36^ reported that higher energy intake leads to higher weight gain, as calf ADG has the highest correlation with total energy intake. It should be noted that although ML-fed calves consumed more starter feeds in the wk before weaning (4 to 6 wk), their growth performance was lower than calves fed HL. This is because the energy intake from the starter feed is not sufficient to compensate for the energy consumption by higher amounts of milk^37^. Our results on weaning weight are in agreement with those of Jafari et al.^22^, where calves were given 4 or 10 L of milk per day and 8 weeks as weaning age. Calves fed a high volume of milk, as in this study, can be expected to increase their ADG by approximately > 600 g per day until weaning^22,38,39^.

The literature on the effects of milk feeding on ADG is inconclusive. Some studies showed higher ADG in calves fed moderate amounts MR or high amounts MR than in calves fed low amounts MR (0.66 and 0.77 vs. 0.55 kg MR /d, DM)^36^ or tended to show higher ADG in calves fed high amounts MR compared to low amounts MR (1.382 vs. 0.691 kg MR /d, DM)^7^. However, some other studies reported that feeding a moderate amount of milk compared with a high amount of milk (315 vs. 409 l/60 or 75 d)^40^ or a low amount of milk compared with a high amount of milk^42^ (210 vs. 370 l/56 d) to dairy calves throughout the study (1–90 and 70 d, respectively) had no effect on ADG. According to Orellana Rivas et al.^36^, calves fed moderate and high amounts of MR (0.66 and 0.77 kg of MR /d) in the preweaning period had similar ADG and skeletal growth.

For structural measurements, we observed a tendency for wither height to increase only in calves fed HL compared to calves fed ML, which may be related to the higher DM intake and ADG and BW of these calves. Quigley et al.^6^ and Dennis et al.^38^ found no change in skeletal growth in calves fed high MR. However, Yohe et al.^7^ (1.382 vs. 0.691 kg MR /d, based on DM) and Jafari et al.^22^ (10 vs. 4 l/d) observed greater hip height and heart girth and a tendency to increase body depth and heart girth in the enhanced plan of liquid fed calves, respectively.

In agreement with the previous reports^7,22,36^, plasma glucose concentration decreased with age in all calves regardless of treatment, which could be related to the physiological change of the primary energy source from glucose to VFA when the rumen becomes functional in young calves^29^. The result of our study is that calves fed HL may have absorbed more lactose through milk at 42 d of age, i.e., during the peak of milk intake, and therefore had a higher glucose concentration, which is in line with previous reports^7,22^. In the study by Orellana Rivas et al.^36^, glucose concentration was not affected by different amounts of MR (0.55, 0.66, and 0.77 kg MR /d, % DM), but insulin concentration increased with higher milk feeding because feeding large amounts of MR increases the sensitivity of the pancreas to insulin release^20,41^. According to the current study, plasma insulin concentrations tended to be higher at 42 d of age in calves fed HL than in calves fed ML, although no differences were observed at 70 d of age. Previous studies have shown that higher plasma glucose concentrations in calves fed HL from MR increase insulin concentrations^5,42^, with the insulin response generally preventing hyperglycemia by glucose excretion^7,41^.

No differences in plasma BHB levels were found between treatments at 42 and 70 d of age. Some studies have shown that feeding large amounts of milk twice daily may delay the development of rumen due to reduced intake of starter feeds^7,18^. In the current study, there were no differences between treatments in starter feed intake and BHB concentrations (an indicator of rumen wall metabolic function) throughout the period, indicating similar rumen development, consistent with rumen fermentation results. In a study by Parsons et al.^39^ calves fed low amounts of milk had higher BHB concentrations than calves fed high amounts of milk at d 36 and 57 (weaning = 63 days), but not at d 70 and 77. In the current study, blood concentrations of BUN and BHB increased with calf age. Consistent with previous studies^7,22,39^, the increase in BHB concentration with calf age was mainly due to the increase in solid feed intake and rumen growth^7^ and also indicates that the animals adapted rapidly to their diet after weaning^43^. In the study by Silva et al.^43^, blood BHB concentrations of calves fed high levels of MR were less than 0.05 mmol/dl before weaning (weaning age = 56 d), but blood BHB concentrations increased rapidly after weaning (0.25 mmol/dl). In addition, higher BUN concentrations in older calves have been reported to be related to a higher intake of crude protein (CP) from solid feed and subsequent breakdown in the rumen^44,45^.

Because early calf feeding and development can affect their later performance, we followed heifer calf performance until the end of the first lactation, although the sample size was small. The results of this study showed that FF had no effect on long-term outcomes, while calves fed HL before weaning tended to be 12.5 kg heavier at first AI and were inseminated 7 d earlier than calves fed ML. Results from Stefanska et al.^46^ showed that a higher growth rate during weaning was associated with lower age at first AI service, gestation, and calving. For all treatments, mean height at first AI and age at calving were 132 cm and 23.3 months, respectively, with no statistically significant differences from FF or milk feeding. Drackley et al.^47^ reported no difference in age to first calving between heifers fed conventional (22% CP, 20% fat) or intensive (28% CP, 20% fat) diets as calves fed MR. However, in the previous studies by Raeth-Knight et al.^1^, and Davis et al.^48^, heifers fed with a high level of milk calved earlier (17, 27.5, and 14 d, respectively) than restricted feeding groups. Korst et al.^11^ reported that age at first calving was not different between the ad libitum and restricted feeding groups. However, milk yield (305 d) during the first lactation was numerically, but not statistically, higher in ad libitum-fed heifers than in restrictive-fed heifers MR^11^. In the study by Keizebrin et al.^50^ age at first calving, BW after first calving, or milk yield and composition during the first lactation was not affected by the amount of milk fed to dairy calves (4 vs. 8 l/d).

During the first lactation, fat and protein yield (305-d), and lactation length were not affected by the treatments, whereas milk yield (305-d) and ECM-305 d of HL-fed calves tended to be 1,195 ± 556 kg and 1052 ± 593 kg higher, respectively, than those of ML-fed calves. It was reported that heifer calves that consumed more nutrients before weaning showed faster growth, better mammary gland development, and higher production^23^. Some studies have examined the effects of high milk or MR volume of dairy calves on milk production in the first lactation and have reported that higher nutritional levels early in life^49^ or higher ADG of calves before weaning^11,23,46^ are associated with greater potential milk yield in the first lactation and higher fat and protein yield. A minimal effect of preweaning ADG on milk production was shown when the growth rate was < 0.5 kg/d^23^ or 0.45 to 0.57 kg/d at 6 wk^10^, but it had a greater effect when the growth rate increased from 0.5 to 0.9 kg/d^23^ or ADG increased from 0.68 to 0.80 kg/d at 6 wk^10^. In the present study, the ADG of calves fed HL was 70 g/d higher than that of calves fed ML (696 and 626 g/d, respectively). In contrast to studies that reported the long-term benefits of accelerated feeding, Aikman et al.^50^ observed no significant effects of ad libitum feeding versus restricted MR (4 l/day) on milk yield, age at first calving, or live weight at first calving. Our results were consistent with our original hypothesis that higher milk feeding has positive effects on growth rate in infancy and consequently on milk yield later in life, although our results tended to be significant and the limited sample size must be considered.

### Effects of feeding frequency

We hypothesized that increasing the frequency of milk feeding in calves fed a higher volume of milk would improve growth performance before weaning and subsequently improve long-term effects on calves. Previous studies have shown that increasing the amount of milk-fed or MR increases the reflux of milk into the reticulorumen and decreases the abomasal emptying rate^19,20^. Thus, increasing the frequency of milk feeding may reduce meal size, accelerate abomasal emptying^19^, better regulate the rate of nutrient entry into the small intestine, reduce energy loss during abomasal fermentation of carbohydrates, and thereby improve nutrient utilization and efficiency^16,19^. However, our result showed no effect of FF or interaction between frequency and milk feeding level on growth performance, blood metabolites, rumen fermentation, and long-term effects during first insemination and lactation. This lack of difference is likely due to a large ability of the abomasum to elongate^17^. Indeed, Ellingsen et al.^17^ showed that the abomasum of calves has a large ability to tolerate distension and calves can consume large amounts of milk (up to 6.8 L/meal) in one meal without risk of milk entering the rumen and causing negative health effects. Overall, the results indicate that increased milk volume has a greater impact on the short- and long-term performance of heifer calves than a small increase in meal size.

## Conclusion

The results of this study indicate that the amount of milk intake has a greater effect on the performance of heifer calves than the frequency of milk feeding. Increasing the amount of milk increased intake and growth performance before weaning, regardless of the frequency of milk feeding. Increases in blood glucose and insulin concentrations prior to weaning were noted in heifers fed higher levels of feed. In addition, calves fed HL were heavier at their first AI, and inseminated at a younger age than calves fed ML, and they tended to produce more milk during their first lactation than calves fed ML. Overall, feeding high levels of milk with the SUSD method improved calf growth performance before weaning and had a positive effect on calf maturation and lactation milk yield.

## Data Availability

The datasets used and/or analyzed during the current study are available from the corresponding author on reasonable request.
